# Differentially private density estimation with skew-normal mixtures model

**DOI:** 10.1038/s41598-021-90276-6

**Published:** 2021-05-26

**Authors:** Weisan Wu

**Affiliations:** 1 Key Laboratory for Applied Statistics of MOE and School of Mathematics and Statistics, Northeast Normal University, Changchun, 130024 China; 2 School of Mathematics and Statistics, Baicheng Normal University, Baicheng, 137000 China

**Keywords:** Statistics, Scientific data

## Abstract

The protection of private data is a hot research issue in the era of big data. Differential privacy is a strong privacy guarantees in data analysis. In this paper, we propose DP-MSNM, a parametric density estimation algorithm using multivariate skew-normal mixtures (MSNM) model to differential privacy. MSNM can solve the asymmetric problem of data sets, and it is could approximate any distribution through expectation–maximization (EM) algorithm. In this model, we add two extra steps on the estimated parameters in the M step of each iteration. The first step is adding calibrated noise to the estimated parameters based on Laplacian mechanism. The second step is post-processes those noisy parameters to ensure their intrinsic characteristics based on the theory of vector normalize and positive semi definition matrix. Extensive experiments using both real data sets evaluate the performance of DP-MSNM, and demonstrate that the proposed method outperforms DPGMM.

## Introduction

With the rapid developments of big data technology, data privacy protection has become a important issue. Differential privacy is an attack-resistant model with strict theoretical proof and good quantitative representation. Differential private density estimation has been done on mixtures model. The most of them are considered in the Gaussian mixtures model (GMM)^1,2^, but the data can presents skewness or heavy tailed behavior. Nearly, the vulnerability of pre-trained models to the attack is not uniform when the training data itself is skewed, Trues et al.^3^ shows that risk from some attacks is routinely increased when models use skewed training data. This risk may be caused by the reduction of the accuracy of the model density estimation on assumed data normality.

To the best of our knowledge no existing work has concern in the skew-normal mixtures model with differential privacy. In skew-normal mixtures model, all data are assumed to be drawn by randomly sampling from one of component of the skew-normal mixture model, and the learning task is to estimate the proportion parameters, location parameters, scale parameters and skewness parameters of each component distribution in model. In particular, we need to consider some problem of skew-normal mixtures model, where (1) skew-normal distribution compose the skew-normal mixtures model, and (2) we not clear the sample belongs to which component, and (3) we just only have perturbed data, and (4) we hope to get estimate values of all parameters.

Addressing on these problems, we propose DP-MSNM, which appends two extra steps (noise adding step and post-processing step) to the general method of density estimation with MSNM in each iteration like. The noise adding step is adding classical Laplace noise to the original estimated parameters in each iteration to achieve differential privacy. The post-processing step will through eigen decomposition on scale parameter to prevent its not positive semi-definite and use normalize method to ensure sum the weight parameter as to 1.

The remainder of the paper is organized as follows. Section ”Preliminaries” introduces preliminaries. Given DP-MSNM details in Section “Differentially private MSNM”. Section “Experimental setup” evaluates the experimental performance. Finally , Section “Conclusions” concludes the work and discusses future works.

## Preliminaries

Let *D* be a data set that contains n records $x_{1},\ldots ,x_{n}$ with *p* attributes, and $x_{i}=(x_{i1},\ldots ,x_{ip})$. There $\Vert x\Vert _{p},\Vert A\Vert _{p}$ are denote the $L_{p}$ norms of vector *x* and matrix *A*.

### Differential privacy

The privacy definition is due to Dwork et al.^4^. The inform definition is given follows.

#### **Definition 1**

(*Differential privacy*) A randomized algorithm mechanism ${\mathscr {M}}$ provides $\varepsilon$-differential privacy, if for all Output *O* of ${\mathscr {M}}$ and for any two neighbor data sets *D* and $D'$, we have

$$\begin{aligned} P({\mathscr {M}}(D)=O) \le e^\varepsilon P({\mathscr {M}}(D')=O) \end{aligned}$$

We called $\varepsilon$ as privacy budget, the smaller $\varepsilon$ means the higher level of privacy provides. Where we adding calibrated random noise to the output to achieved privacy. We use Laplacian mechanism and $L_{1}$-sensitivity to output perturbation.

#### **Definition 2**

($L_{1}$-*sensitivity*) The $L_{1}$ sensitivity of a query function ${\mathscr {M}}$ as the maximum change in the $L_{1}$-norm of the query output on the neighbor data sets *D* and $D'$. That is ,

$$\begin{aligned} S({\mathscr {M}}) = \max _{d(D,D')=1}\Vert {\mathscr {M}}(D)-{\mathscr {M}}(D')\Vert _{1} \end{aligned}$$

where we use Hamming distance to measure the distance between two data sets *D* and $D'$, and its distance is expressed as $d(D,D')=\{i:x_{i} \ne y_{i},x_{i}\in D , y_{i}\in D'\}$. We denote the noisy vector or matrix are follows Laplace distribution $Lap(0,\frac{S({\mathscr {M}})}{\varepsilon })$, where $\frac{S({\mathscr {M}})}{\varepsilon }$ is scale parameter. Given $L_{1}$ sensitivity $S({\mathscr {M}})$ and the privacy budget $\varepsilon$ , this mechanism can ensure differential privacy.

### Density estimation with MSNM

In the multivariate skew-normal mixture models (MSNM), we assumed sample $x_{i}$ is i.i.d. with a density $f_{MSN}(x_{i})$ that can be written as a linear composition of skew-normal distributions^5^ in the form

1 $$\begin{aligned} \begin{aligned} f_{MSNM}(x; \Theta ) =&\sum _{k=1}^{G}\pi _{k} f_{MSN}(x; \theta _{k})\\ =&\sum _{k=1}^{G}\pi _{k}\big \{2\phi ^{d}\big (x;\xi _{k},\Sigma _{k},\alpha _{k}\big ) \Phi \big (\alpha _{k} ^ T\sigma ^{-1}\big (x-\xi _{k}\big )\big )\big \}, \end{aligned} \end{aligned}$$

where $x=(x_{1},\ldots ,x_{n}),$ and $x_{i},i=1,\ldots ,n$ is a *p*-dimensional vector. $\Theta =(\theta _{1},\ldots ,\theta _{G}),\theta _{k}=(\xi _{k},\Sigma _{k}, \alpha _{k}),k=1,\ldots ,G$. where $\pi _{k} \ge 0$ is mixture proportion and satisfied that $\sum _{k=1}^G\pi _{k} =1$. $\xi _{k},\Sigma _{k},\alpha _{k}$ are parameter in the *k*-th component of the mixture models, and $\alpha _{k}$ is the *p*-dimensional skewness parameter, $\Sigma _{k}$ is a $p \times p$ positive semi-definite(PSD) correlation matrix, $\xi _{k}$ is the *p*-dimensional local parameter. $\phi _{d}(x;\xi , \Sigma )$ is the density of a normal distribution $N(\xi , \Sigma )$, $\Phi (\cdot )$ is the cumulate distribution function of standard normal. The function $f_{MSN}(x; \xi ,\Sigma ,\alpha )$ can be present by

2 $$\begin{aligned} f_{MSN}(x; \xi ,\Sigma ,\alpha )=2\phi _{d}(x;\xi ,\Sigma ,\alpha ) \Phi \big (\alpha ^ T\sigma ^{-1}(x-\xi )\big ) \end{aligned}$$

where $\sigma =(\Sigma \odot I_{p})^{\frac{1}{2}}$, $\odot$ denote Hadamard product. If a random variable *X* has density function like Eq. (2), we called the variable X is a Multivariate skew-normal distribution, and denote $X \sim SN_{p}(\xi , \Sigma , \alpha )$ like as Contreras-Reyes and Cortés, 2016^6^.

Recalling the variables $X_{0} \sim SN_{p}(0,{\bar{\Sigma }},\alpha )$ and $X\sim SN_{p}(\xi ,\Sigma ,\alpha )$, we can obtain an additional representation for multivariate skew-normal distribution from the results of Azzalini et al.^5^ as follows:

3 $$\begin{aligned} X_{0} = D_{\delta } Z_{0}+\delta |Z_{1}|, \end{aligned}$$

where $\delta = (\delta _{1}, \delta _{2}, \ldots , \delta _{p})^{T}$ is a vector with the elements in $(-1, 1)$, and

$$\begin{aligned} D_{\delta } = \big (I_{p} - {\text {diag}}(\delta )^{2}\big )^{1/2}, \end{aligned}$$

$U_{0}$ and $U_{1}$ are the normal variables of dimension *p* and 1, the joint distribution is

$$\begin{aligned} \left( \begin{array}{c} Z_{0}\\ Z_{1} \end{array} \right) \sim N_{p+1}\left( 0,\left( \begin{array}{cc} {\bar{\Omega }}&{}\quad 0\\ 0&{}\quad 1 \end{array} \right) \right) , \end{aligned}$$

${\bar{\Psi }}$ is a full-rank correlation matrix.

By the transformation $X = \xi + \sigma X_{0}$, we have

$$\begin{aligned} Y=\xi +\sigma X_{0}=\xi +\sigma D_{\delta } Z_{0}+\sigma \delta |Z_{1}|. \end{aligned}$$

Let $|Z_{1}|=\tau$. Given $\tau$, we can obtain the condition representation, that is,

4 $$\begin{aligned} X|\tau \sim N_{p}(\xi +\sigma \delta \tau ,~\sigma D_{\delta } {\bar{\Omega }} D_{\delta } \sigma ). \end{aligned}$$

For a *p*-dimensional SN distribution, the parameters have the following relationships by simple algebraic work (See^5^):

5 $$\begin{aligned} \omega= & {} D_{\delta }^{-1}\delta ;\nonumber \\ {\bar{\Sigma }}= & {} D_{\delta }\big ({\bar{\Omega }} +\omega \omega ^{T}\big )D_{\delta };\nonumber \\ \alpha= & {} \big (1+\omega ^{T}{\bar{\Omega }}^{-1}\omega \big )^{-1/2}D_{\delta }^{-1}{\bar{\Omega }}^{-1}\omega ;\nonumber \\ \delta= & {} \big (1+\alpha ^{T}{\bar{\Sigma }}\alpha \big )^{-1/2}{\bar{\Sigma }}\alpha . \end{aligned}$$

Furthermore, we have

$$\begin{aligned} {\bar{\Sigma }} = D_{\delta } {\bar{\Omega }}D_{\delta }+D_{\delta } \omega \omega ^{T}D_{\delta } = D_{\delta } {\bar{\Omega }}D_{\delta }+\delta \delta ^{T}. \end{aligned}$$

Thus, let $\eta =\sigma \delta$, by the relation between $\Sigma$ and ${\bar{\Sigma }}$, we have

$$\begin{aligned} \Sigma = \sigma {\bar{\Sigma }}\sigma = \sigma D_{\delta } {\bar{\Omega }}D_{\delta } \sigma + \sigma \delta \delta ^{T}\sigma = \sigma D_{\delta } {\bar{\Omega }}D_{\delta } \sigma + \eta \eta ^{T}. \end{aligned}$$

Therefore

$$\begin{aligned} X|\tau \sim N_{p}\big (\xi +\sigma \delta \tau ,~\Sigma -\eta \eta ^{T}\big ). \end{aligned}$$

Let $Z_{ik}$ be the membership indicator variable such that it equals 1 when $x_{i}$ is from the *k*-th component of the MSNM model, and equals 0 otherwise. Consider the complete data $(X,Z)=\{X_{i},Z_{i}\}_{i=1}^n$, where the latent component-indicators vector $Z_{i}=(Z_{1i},\ldots ,Z_{Gi})$ follows a multi-nomial distribution with the trial and cell probabilities of $\pi _{1},\ldots ,\pi _{G}$. Let us write it as $Z_{i}\sim M(1;\pi _{1},\ldots ,\pi _{G})$. Based on the component indicators, for each $X_{i}$, $i=1,\ldots ,n$, the hierarchical representation for MSNMs can be given by Cabral et al.^7^

6 $$\begin{aligned} X_{i}|\tau _{i},Z_{ik}= & {} 1\sim N_{p}\big (\xi _{k} +\sigma _{k}\delta _{k}\tau _{i},\Sigma _{k}-\eta _{k}\eta _{k}^{T}\big ),\nonumber \\ \tau _{i}|Z_{ik}= & {} 1\sim TN_{[0,+\infty )}(0,1),\nonumber \\&Z_{i}\sim Multi(1;\pi _{1},\ldots ,\pi _{G}), \end{aligned}$$

where $TN_{[0,+\infty )}(0,1)$ denotes the half normal distribution. According to the hierarchical representation (6), ignoring the added constants and denoting

7 $$\begin{aligned} \Lambda _{k}=\Sigma _{k}-\eta _{k}\eta _{k}^{T} \end{aligned}$$

the complete data log-likelihood function is

$$\begin{aligned} \begin{aligned} p\ell _{n}^c(\Theta )&= \sum _{i=1}^{n}\sum _{k=1}^{G}Z_{ij}\Bigg \{\log (\pi _{k})-\frac{1}{2}\log |\Lambda _{k}|-\frac{1}{2}(x_{i}-\xi _{k})^{T}\Lambda _{k}^{-1}(x_{i}-\xi _{k})\\&\quad +(x_{i}-\xi _{k})^{T}\Lambda _{k}^{-1}\eta _{k}\tau _{i}-\frac{1}{2}\eta _{k}^{T}\Lambda _{k}^{-1}\eta _{k}\tau _{i}^{2}-\frac{1}{2}\tau _{i}^{2}\Bigg \} \end{aligned} \end{aligned}$$

According to the Bayesian theorem, we have

$$\begin{aligned} \tau _{i}|(X_{i}=x_{i},Z_{ik}=1)\sim TN_{[0,+\infty )}\big (\mu _{\tau _{ik}},\sigma _{\tau _{ik}}^{2}\big ), \end{aligned}$$

where

$$\begin{aligned} \mu _{\tau _{ik}}=\frac{(x_{i}-\xi _{k})^{T}\Lambda _{k}^{-1}\eta _{k}}{1+\eta _{k}^{T}\Lambda _{k}^{-1}\eta _{k}},~~\sigma _{\tau _{ik}}^{2}=\frac{1}{1+\eta _{k}^{T}\Lambda _{k}^{-1}\eta _{k}}. \end{aligned}$$

Thus, for the current parameters $\Theta ^{(t)}$, let

$$\begin{aligned} \mu _{\tau _{ik}}^{(t)}=\frac{\big (x_{i}-\xi _{k}^{(t)}\big )^{T}\big (\Lambda _{k}^{(t)}\big )^{-1}\eta _{k}^{(t)}}{1+\eta _{k}^{T}\big (\Lambda _{k}^{(t)}\big )^{-1}\eta _{k}^{(t)}},~~ \sigma _{\tau _{ik}}^{2(t)}=\frac{1}{1+\big (\eta _{k}^{(t)}\big )^{T}\big (\Lambda _{k}^{(t)}\big )^{-1}\eta _{k}^{(t)}}. \end{aligned}$$

The EM algorithm then proceeds as follows: *E-step*: Let us compute the conditional expectations as

$$\begin{aligned} \begin{aligned} r_{0ik}^{(t)}&=\frac{\pi _{k}^{(t)}f_{MSN}\big (x_{i};\xi _{k}^{(t)},\Sigma _{k}^{(t)},\alpha _{k}^{(t)}\big )}{\sum _{j=1}^{G}\pi _{j}^{(t)}f_{MSN}\big (x_{i};\xi _{j}^{(t)},\Sigma _{j}^{(t)},\alpha _{j}^{(t)}\big )},\\ r_{1ik}^{(t)}&=E\big (\tau _{i}|X_{i}=x_{i},Z_{ik}=1,\Theta ^{(t)}\big )=\mu _{\tau _{ik}}^{(t)}+\sigma _{\tau _{ik}}^{(t)}\Delta _{ik}^{(t)},\\ r_{2ik}^{(t)}&=E\big (\tau _{i}^{2}|X_{i}=x_{i},Z_{ik}=1,\Theta ^{(t)}\big )= \mu _{\tau _{ik}}^{2(t)}+\sigma _{\tau _{ik}}^{2(t)}+\mu _{\tau _{ik}}^{(t)}\sigma _{\tau _{ik}}^{(t)}\Delta _{ik}^{(t)}, \end{aligned} \end{aligned}$$

where $\Delta _{ik}^{(t)}=\phi \left( \dfrac{\mu _{\tau _{ik}}^{(t)}}{\sigma _{\tau _{ik}}^{(t)}}\right) \bigg / \Phi \left( \dfrac{\mu _{\tau _{ik}}^{(t)}}{\sigma _{\tau _{ik}}^{(t)}}\right)$. Thus, we get $E(Z_{ik}\tau _{i}|X_{i}=x_{i},\Theta ^{(t)})=r_{0ik}^{(t)}r_{1ik}^{(t)},$ and $E(Z_{ik}\tau _{i}^{2}|X_{i}=x_{i},\Theta ^{(t)})=r_{0ik}^{(t)}r_{2ik}^{(t)}$.

Therefore, the Q function can be written as

$$\begin{aligned} \begin{aligned} Q\big (\Theta ,\Theta ^{(t)}\big )&=\sum _{i=1}^n\sum _{k=1}^Gr_{0ik}^{(t)}\Bigg \{\log (\pi _{k})-\frac{1}{2}\log |\Lambda _{k}|-\frac{1}{2}(x_{i}-\xi _{k})^{T}\Lambda _{k}^{-1}(x_{i}-\xi _{k})\\&\quad +(x_{i}-\xi _{k})^{T}\Lambda _{k}^{-1}\eta _{k}r_{1ik}^{(t)}-\frac{1}{2} \big (1+\eta _{k}^{T}\Lambda _{k}^{-1}\eta _{k}\big )r_{2ik}^{(t)}\Bigg \}. \end{aligned} \end{aligned}$$

*M-Step*: Let us maximize $Q(\Theta ,\Theta ^{(t)})$ with respect to $\Theta$ under the restriction with $\sum _{k=1}^G\pi _{k}=1$.

1. Update $$\pi _{k}^{(t)}$$ by $$\pi _{k}^{(t+1)}=n^{-1}\sum _{i=1}^nr_{0ik}^{(t)}$$.
2. Update $$\xi _{k}^{(t)}$$ by
  8 $$\begin{aligned} \xi _{k}^{(t+1)}=\frac{\sum _{i=1}^nr_{0ik}^{(t)}x_{i}-\eta _{k}^{(t)}\sum _{i=1}^nr_{0ik}^{(t)}r_{1ik}^{(t)}}{\sum _{i=1}^nr_{0ik}^{(t)}}. \end{aligned}$$
3. Update $$\eta _{k}^{(t)}$$ by
  9 $$\begin{aligned} \eta _{k}^{(t+1)}=\frac{\sum _{i=1}^nr_{0ik}^{(t)}\big (x_{i}-\xi _{k}^{(t+1)}\big )r_{1ik}^{(t)}}{\sum _{i=1}^nr_{0ik}^{(t)}}. \end{aligned}$$
4. Update $$\Lambda _{k}^{(t)}$$ by
  10 $$\begin{aligned} \begin{aligned} \Lambda _{k}^{(t+1)}&=\frac{1}{\sum _{i=1}^nr_{0ik}^{(t)}}\left\{ \sum _{i=1}^nr_{0ik}^{(t)}\big (x_{i}-\xi _{k}^{(t+1)}\big )\big (x_{i}-\xi _{k}^{(t+1)}\big )^{T} \right. \\&\quad \left. -2 \eta _{k}^{(t+1)}\sum _{i=1}^nr_{0ik}^{(t)}r_{1ik}^{(t)}\big (x_{i}-\xi _{k}^{(t+1)}\big ) + \eta _{k}^{(t+1)}\sum _{i=1}^nr_{0ik}^{(t)}r_{2ik}^{(t)}\big (\eta _{k}^{(t+1)}\big )^{T}\right\} . \end{aligned} \end{aligned}$$

## Differentially private MSNM

In this section, we present DP-MSNM method which is a differentially private density estimation algorithm with MSNM.

### Main idea

Let *G* denotes the component order of the MSNM. The main idea of DP-MSNM is add to two extra steps after getting the original estimated parameters in the M-step of each iteration. The original estimated parameters include mixture proportion ($\pi$), local parameter vector ($\xi _{k}$), scale parameter matrix ($\Sigma _{k}$) and skewness parameter vector ($\alpha _{k}$) of each Skew-Normal distribution, where $\pi =(\pi _{1},\ldots ,\pi _{G})$ and $k\in \{1,\ldots ,G\}$. First, we need to get noise of each parameter by $L_{1}$-sensitivity and allocated privacy budget, and add these noises to the original estimated parameters. We get noisy parameters ${\bar{\pi }}$ and ${\bar{\xi }}_{k},{\bar{\Lambda }}_{k},{\bar{\eta }}_{k}$ . The second step is post-processes ${\bar{\pi }}$ and ${\bar{\xi }}_{k},{\bar{\Lambda }}_{k},{\bar{\eta }}_{k}$, since the noise added will break some intrinsic characteristics of weight parameter and component parameters, this step will output ${\hat{\pi }}$ and ${\hat{\xi }}_{k},{\hat{\Lambda }}_{k},{\hat{\eta }}_{k}$

### Noise adding step

In this part, we will analyze the $L_{1}$ sensitivities of $\pi _{k},\xi _{k},\Lambda _{k},\eta _{k}$, and we will add calibrated noise to the original estimated parameters. We suppose that $D=\{x_{1},\ldots ,x_{n}\}$ and $D'=\{x_{1},\ldots ,x_{n-1}\}$ are two neighbor data sets,where $D'$ has the same $n-1$ records as $D'$, but not have the *n*-th record. Let $N_{k}=\sum _{i=1}^n r_{0ik}$, we also assume the data set is well-separated^8^ and $N_{k}\ge \frac{n}{2G},k=1,\ldots ,G$. We use differentially private k-means algorithm^9^to get the centers of clusters,and get $\eta _{k}$ form skew parameter $\alpha _{k}$ through Mardia measure method^10^. We denote $S(\pi ),S(\xi ),S(\Lambda ),S(\eta )$ as the $L_{1}$-sensitivities of $\pi ,\xi _{k},\Lambda _{k},\eta _{k}$ in each iteration. According to Eqs. (8)–(10), we give $S(\pi ),S(\xi ),S(\Lambda ),S(\eta )$ in Lemmas 1, 2, 3 and 4.

#### **Lemma 1**

*In each iteration, the* $L_{1}$-*sensitivity of* $\pi$ *is* $S(\pi )=\frac{G}{n}$.

The proof process of Lemma 1 is identical as Wu et al.^1^. In order to make the proof of subsequent lemmas clear, we still write it down as follows.

#### *Proof*

Since $\pi =(\pi _{1},\ldots ,\pi _{G})$, let $\pi (D)$ and $\pi (D')$ denote the weights of data sets *D* and $D'$. Then the k-th elements of $\pi (D)-\pi (D')$ is $\frac{\sum _{i=1}^nr_{0ik}}{n} - \frac{\sum _{i=1}^{n-1}r_{0ik}}{n-1} =\frac{\sum _{i=1}^{n-1}(r_{0nk}-r_{0ik})}{n(n-1)}$. Since $|r_{0nk}-r_{0ik}|<1$, we have $\Vert \pi (D) - \pi (D')\Vert _{1} = \sum _{k=1}^G |\frac{\sum _{i=1}^{n-1}(r_{0nk}-r_{0ik})}{n(n-1)}| \le \sum _{k=1}^G \frac{\sum _{i=1}^{n-1}|r_{0nk}-r_{0ik}|}{n(n-1)}\le \frac{G}{n}$. $\square$

#### **Lemma 2**

*In each iteration, if* $\Vert x_{i}\Vert _{1}\le R,N_{k}\ge \frac{n}{2G}$ *and* $\Vert \eta _{k}\Vert _{1}\le R_{3}$,*the* $L_{1}$-*sensitivity of* $\xi _{k}$ *is* $S(\xi )=\frac{4G(R-R_{3})}{n}$.

#### *Proof*

Let $\xi (D)$ and $\xi (D')$ denote the local parameter of data sets *D* and $D'$. The k-th elements of $\xi (D)-\xi (D')$ is

$$\begin{aligned} & {}\frac{\sum _{i=1}^nr_{0ik}x_{i} - \eta _{k}\sum _{i=1}^nr_{0ik}r_{1ik}}{\sum _{i=1}^nr_{0ik}} - \frac{\sum _{i=1}^{n-1}r_{0ik}x_{i} - \eta _{k}\sum _{i=1}^{n-1}r_{0ik}r_{1ik}}{\sum _{i=1}^{n-1}r_{0ik}}\\&\quad =\frac{r_{0nk}\sum _{i=1}^{n-1}r_{0ik}(x_{n} -x_{i})- \eta _{k}r_{0nk}\sum _{i=1}^nr_{0ik}(r_{1nk}-r_{1ik})}{\sum _{i=1}^nr_{0ik}\sum _{i=1}^{n-1}r_{0ik}} \end{aligned}$$

Because of$\Vert x_{i}\Vert _{1}\le R,N_{k}\ge \frac{n}{2G},\Vert \eta _{k}\Vert _{1}\le R_{3}$, we have

$$\begin{aligned} \begin{aligned} \Vert \xi (D)-\xi (D')\Vert _{1}&\le \frac{2G}{n}\frac{|Rr_{0nk}\sum _{i=1}^{n-1}r_{0ik} - \eta _{k}r_{0nk}\sum _{i=1}^{n-1}r_{0ik}|}{\sum _{i=1}^{n-1}r_{0ik}}\\&\le \frac{2G}{n}(|Rr_{0nk} - \eta _{k}r_{0nk}|)\\&\le \frac{4G(R-R_{3})}{n}. \end{aligned} \end{aligned}$$

#### **Lemma 3**

*In each iteration, if* $\Vert x_{i}\Vert _{1}\le R,N_{k}\ge \frac{n}{2G}$ *and* $\Vert r_{1ik}\Vert _{1}\le R_{1},\Vert r_{2ik}\Vert _{1}\le R_{2}$, *the* $L_{1}$-*sensitivity of* $\eta _{k}$ *is* $S(\eta )=\frac{2GRR_{1}}{nR_{2}}$.

#### *Proof*

let $\eta (D)$ and $\eta (D')$ denote the parameter in the Eq. (9) *D* and $D'$. The k-th elements of $\eta (D)-\eta (D')$ is

$$\begin{aligned} & {} \frac{\sum _{i=1}^nr_{0ik}(x_{i} -\xi _{k})r_{1ik}}{\sum _{i=1}^nr_{0ik}r_{2ik}} - \frac{\sum _{i=1}^{n-1}r_{0ik}(x_{i} -\xi _{k})r_{1ik}}{\sum _{i=1}^{n-1}r_{0ik}r_{2ik}}\\&\quad =\frac{r_{0ik}\sum _{i=1}^{n-1}r_{0ik}[r_{2ik}r_{1ik}(x_{n} -\xi _{k}) - r_{2ik}r_{1ik}(x_{i} -\xi _{k})]}{\sum _{i=1}^nr_{0ik}r_{2ik}\sum _{i=1}^{n-1}r_{0ik}r_{2ik}} \end{aligned}$$

Then

$$\begin{aligned} \begin{aligned} \Vert \eta (D)-\eta (D')\Vert _{1}\le \frac{2GRR_{1}}{nR_{2}}. \end{aligned} \end{aligned}$$

$$\square$$

#### **Lemma 4**

*In each iteration, if* $\Vert x_{i}\Vert _{1}\le R,N_{k}\ge \frac{n}{2G},\Vert r_{1ik}\Vert _{1}\le R_{1},\Vert r_{2ik}\Vert _{1}\le R_{2},\Vert \eta _{k}\Vert _{1}\le R_{3}$, *the* $L_{1}$-*sensitivity of* $\Lambda _{k}$ *is* $S(\Lambda )=\frac{2G(R^{2}+4RR_{1}R_{3}+4R_{2}R_{3}^{2})}{n}$.

#### *Proof*

let $\Lambda (D)$ and $\Lambda (D')$ denote the parameter in the Eq. (10) *D* and $D'$. The k-th element of vector $\Lambda (D)-\Lambda (D')$ is

$$\begin{aligned} & {}\frac{\sum _{i=1}^nr_{0ik}(x_{i} -\xi _{k})(x_{i} -\xi _{k})^{T} - 2\eta _{k}\sum _{i=1}^nr_{0ik}r_{1ik}(x_{i} -\xi _{k})^{T} + \eta _{k}\sum _{i=1}^nr_{0ik}r_{2ik}\eta _{k}^{T}}{\sum _{i=1}^nr_{0ik}}\\&\qquad - \frac{\sum _{i=1}^{n-1}r_{0ik}(x_{i} -\xi _{k})(x_{i} -\xi _{k})^{T} - 2\eta _{k}\sum _{i=1}^{n-1}r_{0ik}r_{1ik}(x_{i} -\xi _{k})^{T} + \eta _{k}\sum _{i=1}^{n-1}r_{0ik}r_{2ik}\eta _{k}^{T}}{\sum _{i=1}^{n-1}r_{0ik}}\\&\quad =\frac{1}{\sum _{i=1}^{n}r_{0ik}\sum _{i=1}^{n-1}r_{0ik}}\left\{ r_{0nk}\sum _{i=1}^{n-1}\left[ (x_{n} -\xi _{k})(x_{n} -\xi _{k})^{T}-(x_{i} -\xi _{k})(x_{i} -\xi _{k})^{T}\right] \right. \\&\qquad \left. -2\eta _{k}r_{0nk}\sum _{i=1}^{n-1}r_{0ik}\left[ r_{1nk}(x_{n} -\xi _{k})^{T} - r_{1ik}(x_{i} -\xi _{k})^{T}\right] + \eta _{k}\sum _{i=1}^{n-1}r_{0ik}\left[ r_{0nk}r_{2nk}\eta _{k}^{T}- r_{0nk}r_{2ik}\eta _{k}^{T}\right] \right\} \end{aligned}$$

Then

$$\begin{aligned} \begin{aligned} \Vert \Lambda (D)-\Lambda (D')\Vert _{1}\le \frac{2G(R^{2}+4RR_{1}R_{3}+4R_{2}R_{3}^{2})}{n}. \end{aligned} \end{aligned}$$

In each iteration, let $\varepsilon _{\pi },\varepsilon _{\xi },\varepsilon _{\Lambda },\varepsilon _{\alpha }$ present the privacy budget allocated to $\pi ,\xi _{k},\Lambda _{k},\alpha _{k}$ respectively, where $k=1,\ldots ,G$. So, we know the privacy budget in each iteration is $\varepsilon _{\pi } + G(\varepsilon _{\xi }+\varepsilon _{\Lambda }+\varepsilon _{\alpha })$ from composite theorem. Next we can get the noisy parameters ${\bar{\pi }},{\bar{\xi }}_{k},{\bar{\Lambda }}_{k},{\bar{\alpha }}_{k}$ through Lemmas 1, 2, 3 and 4 and the Laplace mechanism, it was given in follows:

$$\begin{aligned} \begin{aligned} {\bar{\pi }}_{k}&= \pi _{k} + e_{\pi _{k}}^{1\times 1},\\ {\bar{\xi }}_{k}&= \xi _{k} + e_{\xi _{k}}^{p\times 1},\\ {\bar{\Lambda }}_{k}&= \Lambda _{k} + e_{\Lambda _{k}}^{p\times p},\\ {\bar{\eta }}_{k}&= \eta _{k} + e_{\eta _{k}}^{p\times 1}. \end{aligned} \end{aligned}$$

where $e_{\pi _{k}}\sim Lap(0,\frac{S(\pi )}{\varepsilon _{\pi }}),e_{\xi _{k}}\sim Lap(0,\frac{S(\xi )}{\varepsilon _{\xi }}),e_{\Lambda _{k}}\sim Lap(0,\frac{S(\Lambda )}{\varepsilon _{\Lambda }}),e_{\eta _{k}}\sim Lap(0,\frac{S(\eta )}{\varepsilon _{\eta }})$ denote noise respect to $\pi _{k},\xi _{k},\Lambda _{k},\eta _{k}$.

### Post-processing step

The noise parameters ${\bar{\pi }},{\bar{\xi }}_{k},{\bar{\Lambda }}_{k},{\bar{\eta }}_{k}$ which obtained in the Noise adding step may not satisfy some of the basic properties of the original parameters. Firstly, ${\bar{\pi }}$ not satisfied $\pi _{k} \ge 0$ and $\sum _{k=1}^n =1$ generally. Secondly, ${\bar{\Lambda }}_{k}$ would not be a positive semi-definite matrix after adding noisy. If the noisy covariance matrix ${\bar{\Lambda }}_{k}$ is not PSD matrix, the other parameters, for instance ${\bar{\xi }}_{k},{\bar{\alpha }}_{k}$ can not be calculated exactly. We need to solve these problems as follow.

First, we use the method of normalized to the ${\bar{\pi }}$. We let ${\bar{\pi }}_{k} =\frac{{\bar{\pi }}_{k} -\min \{{\bar{\pi }}\}}{\max \{{\bar{\pi }}\}-\min \{{\bar{\pi }}\}}$, and make them sum to one by ${\hat{\pi }}_{k} =\frac{{\bar{\pi }}_{k} +\zeta }{\sum _{k=1}^G{\bar{\pi }}_{k}+ G\zeta }$. There $\zeta$ is a small number, it can be smooth ${\bar{\pi }}$

Secondly, we can use theory of Higham^11^ to ensure the covariance matrix ${\bar{\Sigma }}_{k}$ to be PSD through post-processing ${\bar{\Lambda }}_{k}$. Like Wu et al.^1^, we use the follow equation

11 $$\begin{aligned} {\hat{\Lambda }}_{k} = {\bar{\Lambda }}_{k} + \varrho ({\bar{\Lambda }}_{k})I, \end{aligned}$$

where $\varrho ({\bar{\Lambda }}_{k}) = \min \{r\ge 0:{\bar{\Lambda }}_{k} +rI \succeq 0\}$, $\succeq 0$ means that all the eigenvalues are not less than 0, *I* is the identity matrix. We use ${\hat{\Lambda }}_{k}$ to get ${\hat{\xi }}_{k}$, and next we will get ${\hat{\eta }}_{k},{\hat{\Sigma }}_{k}$ and ${\hat{\alpha }}_{k}$ by Eqs. (5) and (7). The parameters ${\hat{\xi }}_{k},{\hat{\Sigma }}_{k},{\hat{\alpha }}_{k}$ will be join into the next iteration calculation.

Although adding two extra steps on original parameters, we can know the parameters ${\hat{\pi }},{\hat{\xi }},{\hat{\alpha }},{\hat{\Sigma }}$ also satisfied $\varepsilon$-differential privacy^12^.

### DP-MSNM

From what discussed above, we give the DP-MSNM algorithm in Algorithm 2, where lines 1–2 allocate the privacy budget and initialize the parameters, lines 3 calculates the $L_{1}$-sensitivities of all parameters, lines 5–6 execute the normal E-step and M-step, lines 7–12 execute the noise adding step, lines 13–20 execute the post-processing step. Given total privacy budget $\varepsilon$, Wu et al.^1^shows that under the maximum iteration *T*, Algorithm 2 satisfies $\varepsilon$-differential privacy. for $k=1,\ldots ,G$, we allocate the privacy budget $\varepsilon _{\Lambda } = \frac{0.6}{T*G}\varepsilon$ to $\Lambda _{k}$,$\varepsilon _{\xi } = \frac{0.13}{T*G}\varepsilon$ and $\varepsilon _{\eta } = \frac{0.13}{T*G}\varepsilon$ to $\xi _{k}$ and $\eta _{k}$ respectively, because of the higher $\varepsilon _{\Lambda }$ can reduce the amount of noise added to the $\Lambda _{k}$.

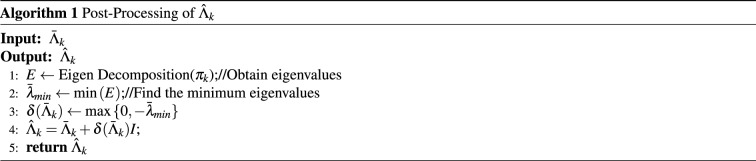

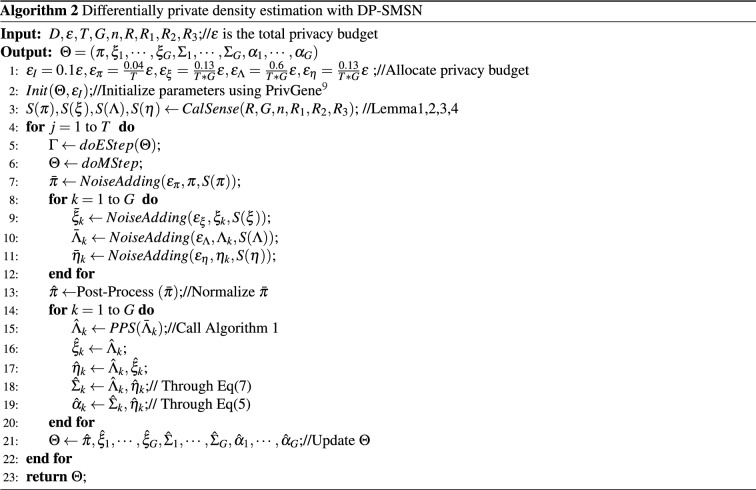





## Experimental evaluation

Our experimental implemented in R version 3.6.3^13^ based on mixsmsn package^7^ to get DP-MSNM results, and all experiments are performed on a laptop with Intel Core i7-9750H and 2.60 GHz CPU and 6.00 RAM.

### Experimental setup

We evaluate DP-MSNM on two real data sets, the Australian Institute of Sport data set and the 3DRoad Network data set, which from UCI Machine Learning Repository. We choose the columns 2–3 in AIS data set and the columns 2–4 in 3DRoad data set. For convenience, we’ll still assume that the default values for both data sets are the same as those in Wu et al.^1^. In this article, we use the complete data set, and the default values are recorded in the Table 1.

**Table 1:** Description of data sets.

	n	d	K	B	T
3DRoad	434,847	3	6	2	30
AIS	202	2	2	2	30

**Figure 1:**
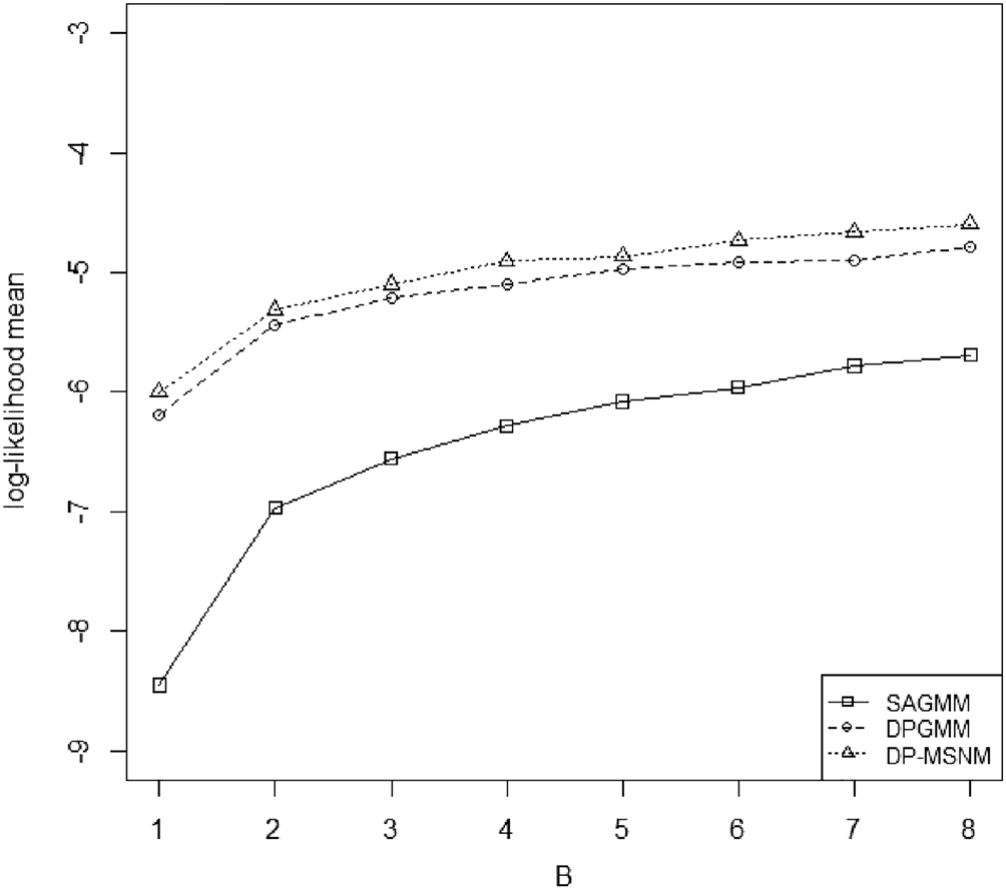
Effect of total privacy budget on AIS data set.

**Figure 2:**
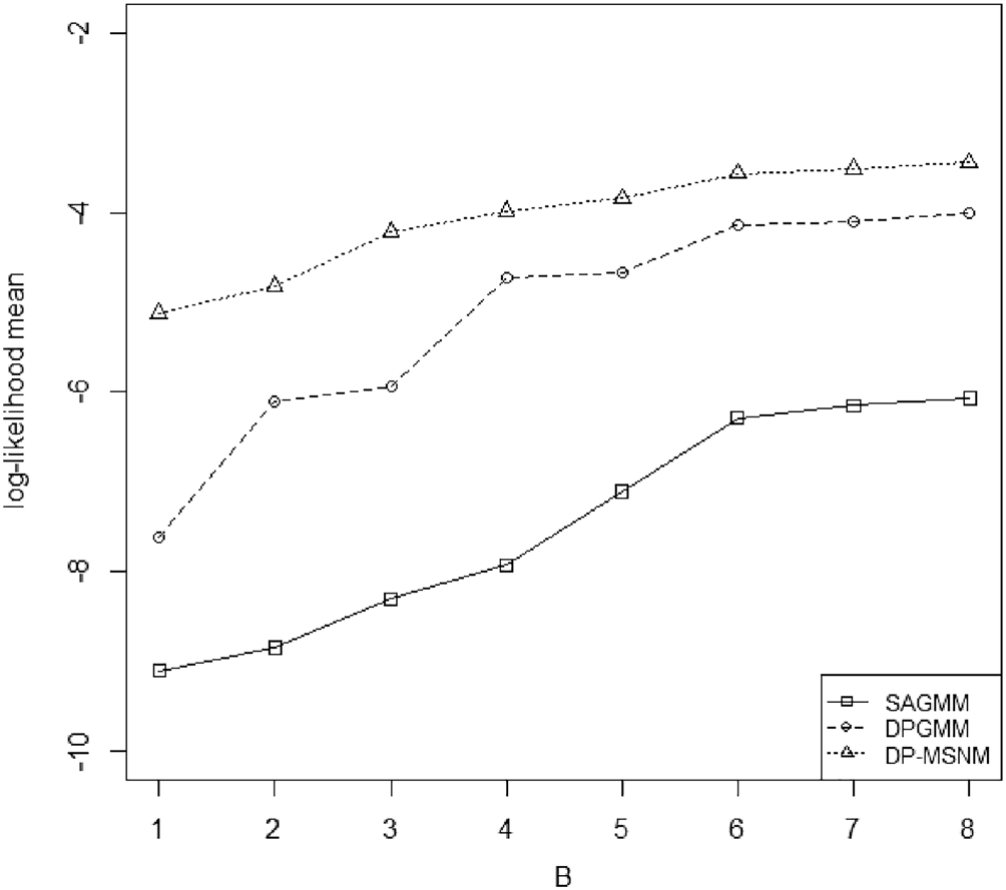
Effect of total privacy budget on 3DRoad data set.

We use log-likelihood mean $\frac{1}{n} \log \{ f_{MSNM}(x;\pi ,\xi ,\Sigma ,\alpha )\}$ which is the average log-likelihood of the whole data set to evaluate the performance of DP-MSNM. A larger logarithmic likelihood mean means that the parameters estimated by the model have a better performance. We compare the density estimation effects of SAGMM, DPGMM and DP-MSNM with log-likelihood mean models for the two data sets respectively (see Figs. 1 and 2). In particular, we use AIC and BIC criteria^6^ to compare the fitting effect of DPGMM algorithm and DP-MSNM algorithm on two data sets.

## Results

### Compare with DP-GMM and SAGMM

We implement SAGMM by applying the privacy preserving platform GUPT . The default values are the same as that of DP-MSNM . For the number of partitions, and the spherical covariance in SAGMM which is assumed known before the experiment. We set the number of partitions as 5. Different variances are applied and the best spherical covariance is chosen for each experiment. From Figs. 1 and 2 we observed that the performance of the three methods improve when B increases, DP-MSNM outperforms SAGMM and DPGMM. In terms of the shape of the curve in Fig. 1, our results are different from those of Wu et al.^1^, mainly because we used a larger amount of data in the data set 3DRoad, and in order not to lose data information, we did not normalized each attribute of the data set.

We implement DPGMM by software R with the same environment as the DP-MSNM. From the Figs. 1 and 2 we find the DP-MSNM outperforms DPGMM.

### Effect of number of skew-normal distribution (G)

For AIS data set, as can be seen from Table 2, using DPGMM algorithm, the values of AIC and BIC are the minimum when k = 3. By using DP-MSNM algorithm, the values of AIC and BIC are the smallest when k = 2. For 3DRoad data set, as can be seen from Table 3, using DPGMM algorithm, the values of AIC and BIC are the minimum when k = 8. By using DP-MSNM algorithm, the values of AIC and BIC are the smallest when k = 7.

**Figure 3:**
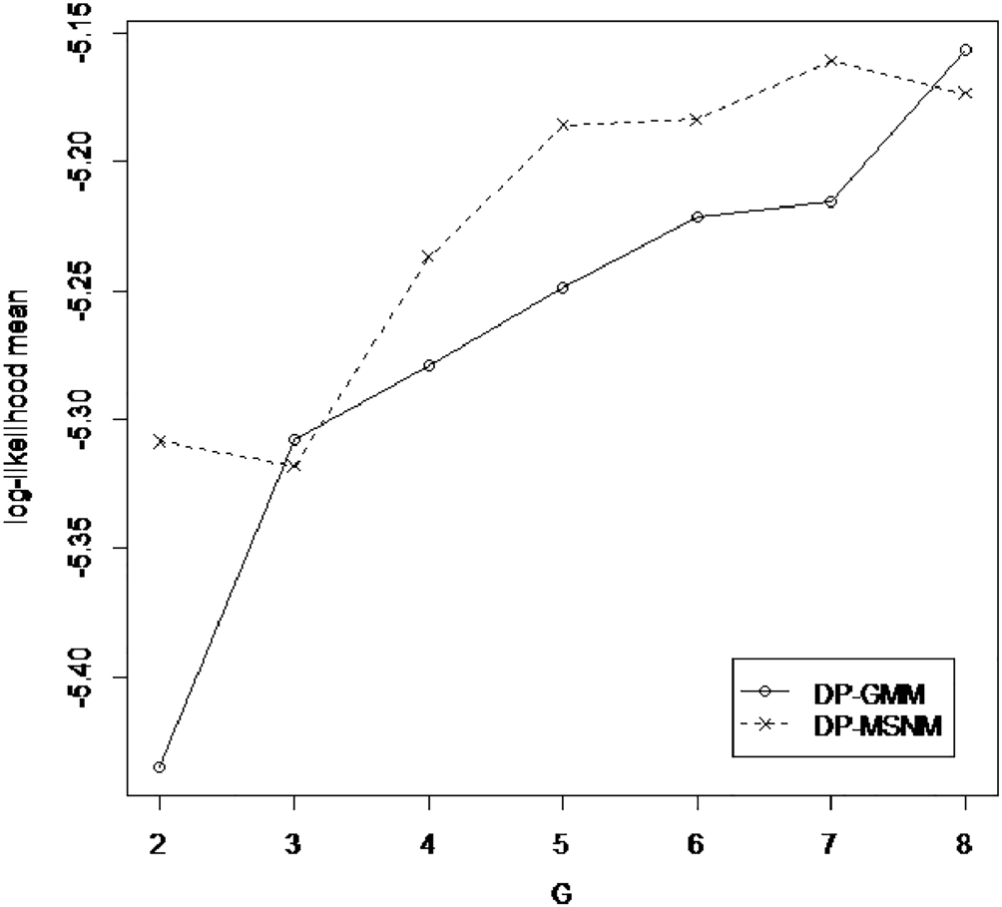
Effect of G in AIS data set.

**Figure 4:**
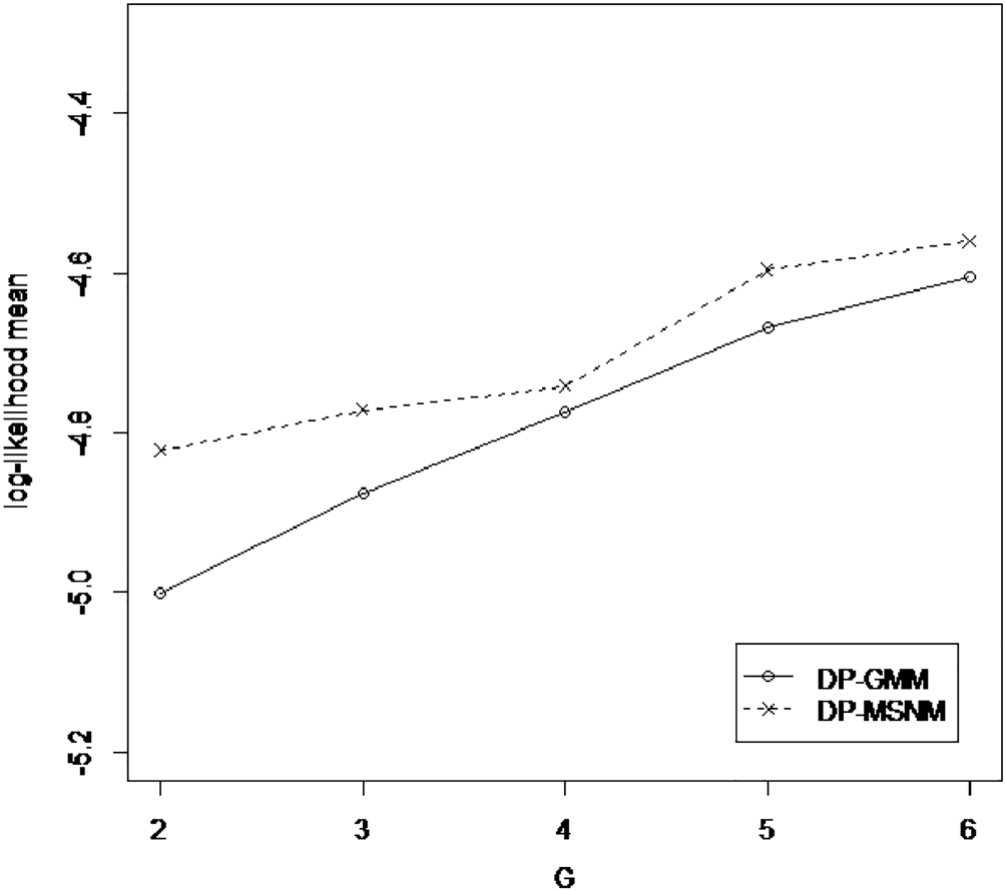
Effect of G in 3DRoad data set.

These results is consistent with the results we can see from Figs. 3 and 4, because for AIS data, the log-likelihood mean of DPGMM algorithm at k = 3 is close to that of DP-MSNM algorithm (Fig. 3).A similar thing happened with 3Droad data (Fig. 4).

So, we can conclude that the order of the component has great effect to our results.

**Table 2:** DP-SMSN algorithm in AIS data set.

k	2	3	4	5	6	7	8
**DPGMM**							
AIC	2217.618	2178.371	2184.419	2187.219	2186.657	2180.705	2188.74
BIC	2254.009	2234.611	2260.509	2283.159	2302.446	2316.344	2344.229
**DP-MSNM**							
AIC	2174.956	2186.836	2204.82	2205.515	2208.153	2195.302	2209.796
BIC	2224.58	2262.926	2307.377	2334.537	2363.642	2377.257	2418.217

For each component $k(k = 2, \ldots , 8)$, the Akaike (AIC) and Bayesian (BIC) information criteria, appear in two models.

**Table 3:** DP-SMSN algorithm in 3DRoad data set.

k	2	3	4	5	6	7	8
**DPGMM**							
AIC	4,350,524	4,242,447	4,103,088	4,057,006	4,006,059	3,957,658	3,882,209
BIC	4,350,733	4,242,766	4,103,516	4,057,544	4,006,707	3,958,415	3,883,077
**DP-MSNM**							
AIC	4,194,876	4,148,930	4,139,086	3,995,200	3,941,379	3,898,330	3,904,047
BIC	4,195,150	4,149,348	4,139,646	3,995,903	3,942,225	3,899,318	3,905,178

For each component $k(k = 2, \ldots , 8)$, the Akaike (AIC) and Bayesian (BIC) information criteria, appear in two models.

## Conclusions

In this paper, we proposed DP-MSNM, which is a effective and accurate privacy-preserving mechanism to estimate density. We added noise to original parameters and through post-processing step to ensure weight and covariance parameter have it’s intrinsic characteristics. In the future, we will extend the DP-MSNM model to high-dimensional data. We also plan to construct other Skew-family distributions to differential privacy.
